# Supplementary material for: Deacylated Derivative of Hericenone C Treated by Lipase Shows Enhanced Neuroprotective Properties Compared to Its Parent Compound
Source: Molecules. 2023 Jun 5;28(11):4549. doi: 10.3390/molecules28114549 (PMC10254140; doi:10.3390/molecules28114549)
Supplement: Supplementary file 1 [file molecules-28-04549-s001.zip › molecules-2426134-supplementary.pdf]

Supplementary data

Deacylated Derivative of Hericenone C Treated by Lipase Shows Enhanced Neuroprotective Properties Compared to Its Parent Compound

Sonam Tamrakar [1,2,†], Dongmei Wang [1,†], Eri Hiraki [1,†], Chunguang Han [1], Yang Ruan [1], Ahmed E. Allam [1,3], Yhiya Amen [4], Yoshinori Katakura [5] and Kuniyoshi Shimizu [1,*]

[1] Department of Agro-Environmental Sciences, Faculty of Agriculture, Kyushu University, Fukuoka 819-0395, Japan; tamraka3@msu.edu (S.T.); spswdm@hotmail.com (D.W.); e.hiraki.835@gmail.com (E.H.); syunkouk@gmail.com (C.H.); ahxcry9619@hotmail.com (Y.R.); ahmedezzaldeanallam@gmail.com (A.E.A.)
[2] Department of Fisheries and Wildlife, College of Agriculture and Natural Resources, Michigan State University, East Lansing, MI 48824, USA
[3] Department of Pharmacognosy, Faculty of Pharmacy, Al-Azhar University, Assiut 71524, Egypt
[4] Department of Pharmacognosy, Faculty of Pharmacy, Mansoura University, Mansoura 35516, Egypt; yhiyaamen@gmail.com
[5] Department of Genetic Resources Technology, Faculty of Agriculture, Kyushu University, Fukuoka 819-0395, Japan; katakura@grt.kyushu-u.ac.jp
* Correspondence: shimizu@agr.kyushu-u.ac.jp
† These authors contributed equally to this work.

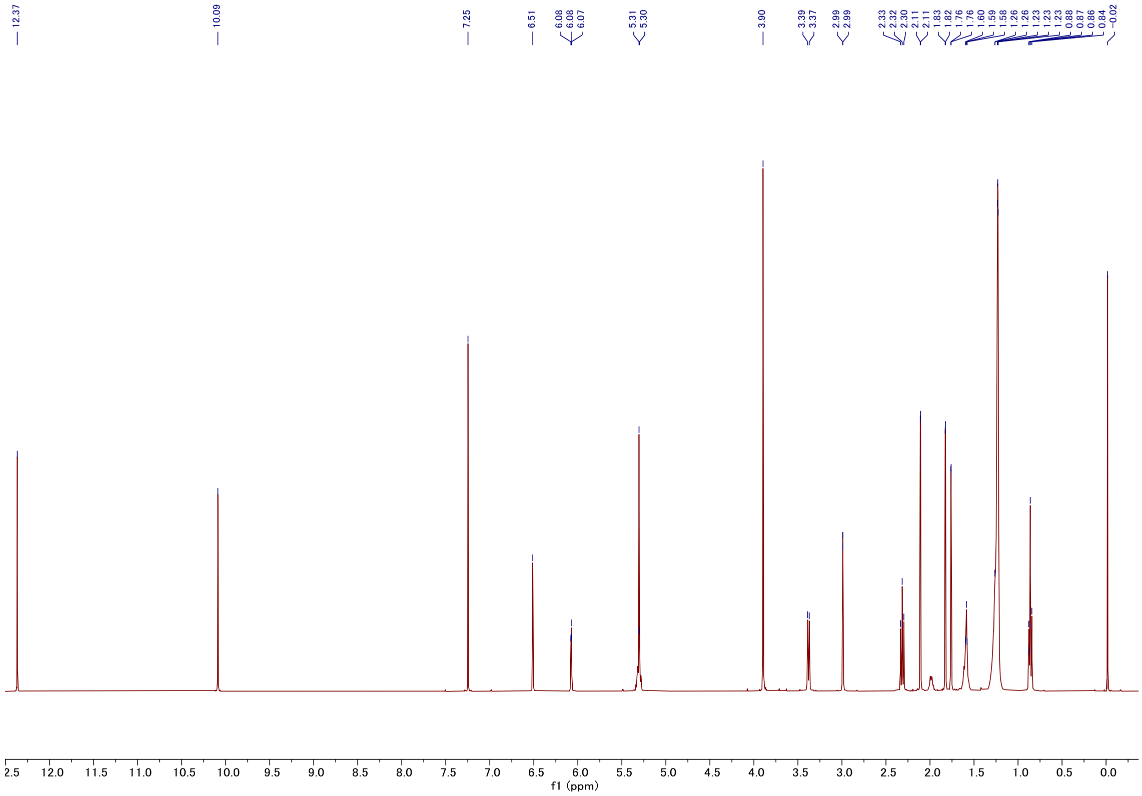

Figure S1. ^{1}H-NMR spectrum of compound **1** ($CDCl_3$, 400 MHz)

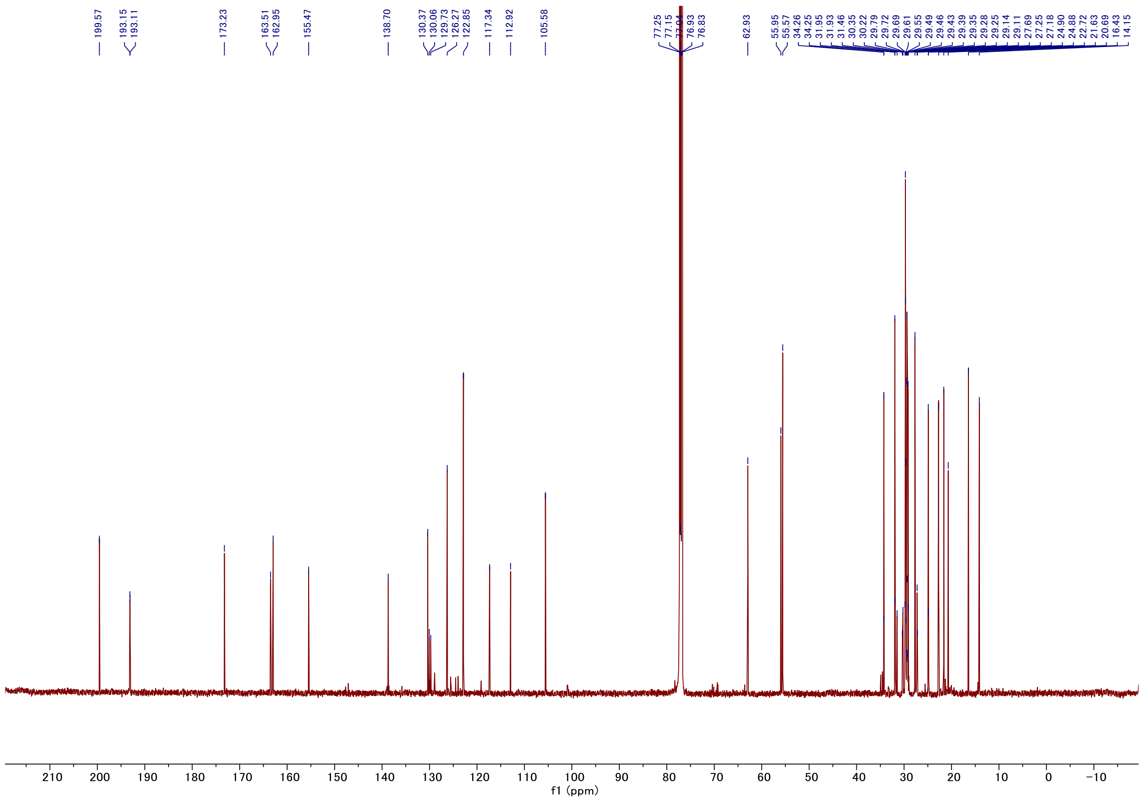

Figure S2. ^{13}C-NMR spectrum of compound **1** ($CDCl_3$, 150 MHz)

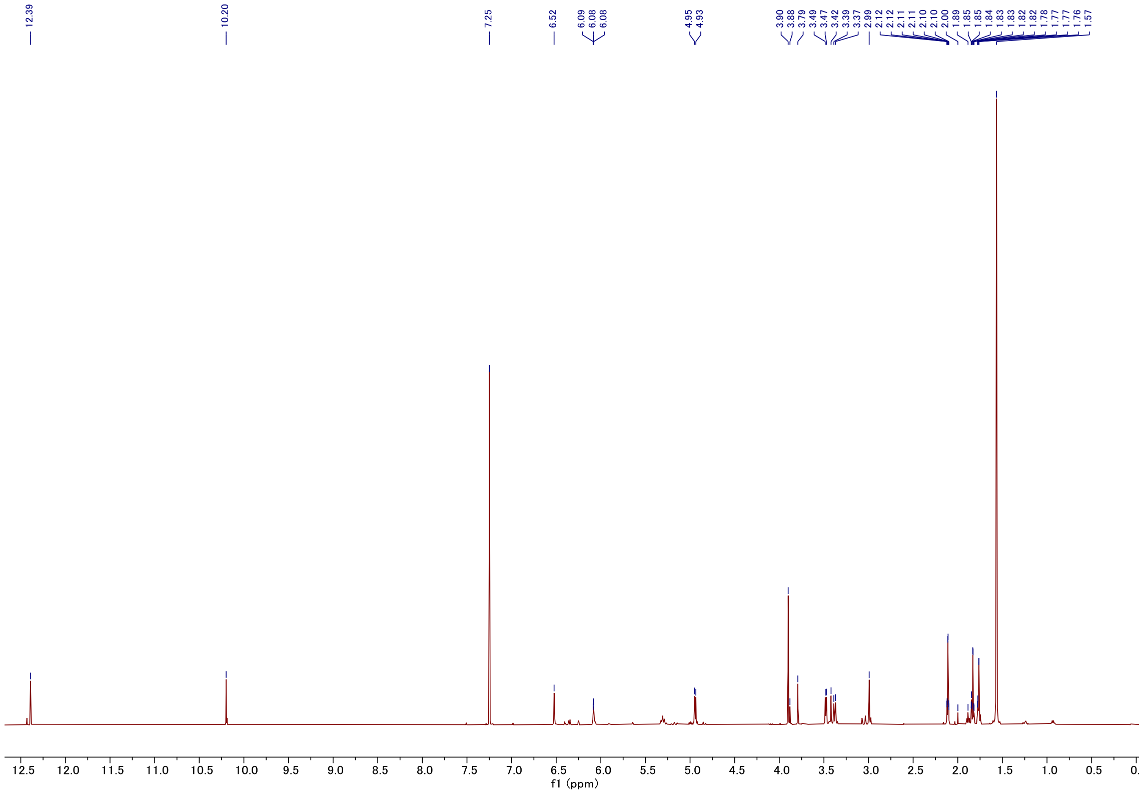

Figure S3. ^{1}H-NMR spectrum of compound **2** ($CDCl_3$, 400 MHz)

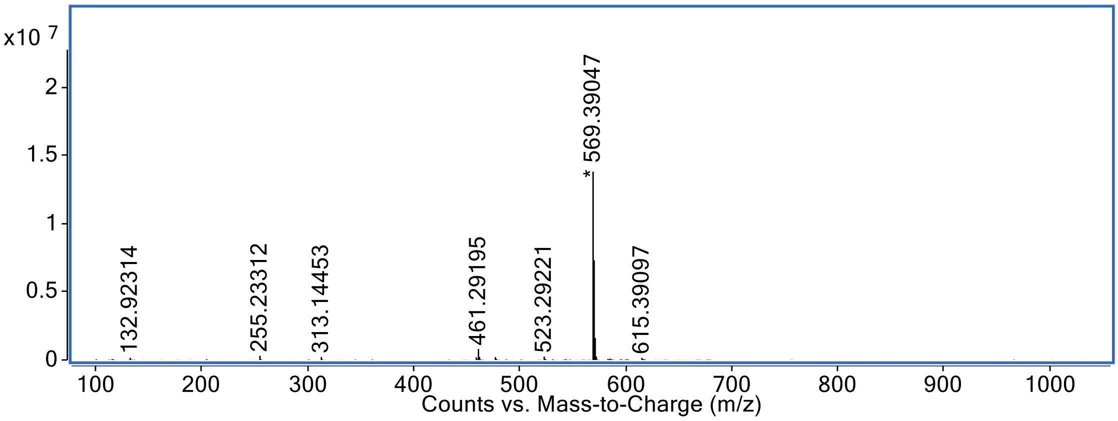

Figure S4. HR-ESI-MS Analysis of compound **1**

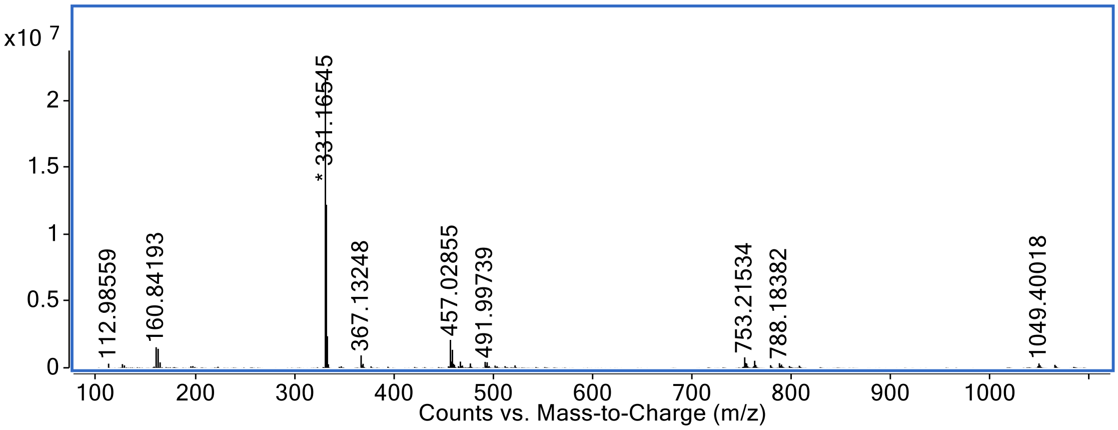

Figure S5. HR-ESI-MS Analysis of compound **2**
